# Identifying the *In Vivo* Cellular Correlates of Antipsychotic Drugs

**DOI:** 10.1523/ENEURO.0220-18.2018

**Published:** 2018-12-07

**Authors:** Radhika S. Joshi, Mitradas M. Panicker

**Affiliations:** 1 National Centre for Biological Sciences (Tata Institute of Fundamental Research), Bengaluru 560065, India

**Keywords:** 5-HT2A receptor knock-out, antipsychotics, c-Fos

## Abstract

GPCRs such as 5-HT_2A_ and D2 are implicated in the therapeutic and the side effects of antipsychotics. However, the pattern of brain activity that leads to the behavioral effects of antipsychotics is poorly understood. To address this question, we used the transgenic ‘FosTRAP’ mice (*Mus musculus*), where a fluorescent reporter marks the cells responsive to the stimulus of interest. Here, the stimulus was an administration of various antipsychotic drugs. In case of typical antipsychotics such as Haloperidol, the *c-fos* active cells were predominantly found in the striatum, whereas in case of the atypical antipsychotics (Clozapine and Olanzapine), *c-fos*-induced cells were more numerous in the cortical regions, e.g., orbital cortex, piriform cortex. Curiously, we also observed ependymal cells to be a novel cellular target of atypical antipsychotics. 5-HT_2A_ is considered to be a major target for atypical antipsychotics. Therefore, we bred ‘FosTRAP’ mice with 5-HT_2A_ knock-out (KO) mice and tested their response to the prototype of atypical antipsychotics, Clozapine. Interestingly, the absence of 5-HT_2A_ did not significantly affect the number of *c-fos*-induced cells in the cortical regions. However, the ependymal cells showed a dramatically reduced response to Clozapine in the absence of 5-HT_2A_. In summary, the TRAP system has allowed us to identify various region-specific activity induced by antipsychotics and novel cellular targets of the antipsychotics. These results serve as a “proof of principle” study that can be extended to explore the biochemical and physiological changes brought about by antipsychotics and specifically identify antipsychotic-responsive cells in the live tissue.

## Significance Statement

Antipsychotic drugs have been the first choice of treatment for mental illnesses such as schizophrenia. Thorough understanding of the cellular and neuronal targets of these drugs should deepen our understanding of the pathophysiology of the mental disorders and help the development of improved therapy. We report here the use of FosTrap mice that allowed us to label cells and brain areas that were responsive to the antipsychotic treatment. We identified brain regions such as orbital cortex, piriform cortex, and ventral-posteromedial thalamus (Vpm) as the targets of the atypical class of antipsychotics. Importantly, we also report for the first time that, ependymal cells, lining the ventricles, are targeted by the atypical antipsychotics and this effect is modulated by the 5-HT_2A_ receptor.

## Introduction

Antipsychotics have revolutionized the treatment of mental illness from the 1950s (Hippius, 1989; Shen, 1992). Even today, antipsychotics are the preferred treatment for schizophrenia (Roth et al., 2004; Ross et al., 2006; Leucht et al., 2009). However, the mechanism of action of antipsychotics still remains unclear and controversial, and the brain areas, neural circuits and cellular targets involved in the effects of antipsychotics need to be better identified.

Binding affinities have suggested the D2 dopamine receptor and the serotonin receptor 5-HT_2A_ as the prime targets of antipsychotics (Roth et al., 1994, 2004; Yadav et al., 2011a). Based on the relative affinities for the 5-HT_2A_ and D2, antipsychotics are also classified as atypical or typical. Typical antipsychotics exhibit higher affinity for D2 than 5-HT_2A_ and the reverse is seen for atypical antipsychotics (Meltzer et al., 1989). Antagonism at the 5-HT_2A_ and D2 is thought to underlie some of the therapeutic effects and/or side effects of antipsychotics (Kapur et al., 1995, 2000; Wadenberg et al., 2000; Fribourg et al., 2011; Moreno et al., 2016). Along with the 5-HT_2A_ and D2, antipsychotics can also bind to many other GPCRs with varying affinities, for example, muscarinic receptors, adrenergic receptors, and histamine receptors (Roth et al., 2004). However, the role of these GPCRs in modulating the antipsychotic-induced effects on neural circuits or cellular targets is largely unknown.

Previously, *c-fos* activity has been used to identify the brain areas and cells that are active on the administration of antipsychotics. Up-regulation of *c-fos* gene has been suggested as a marker for neuronal activity. Many stimuli have been shown to cause up-regulation of *c-fos*, for example, seizure (Morgan et al., 1987; Gunn et al., 1990), membrane depolarization (Sheng et al., 1990), and novel environment and psychoactive drugs (Day et al., 2001; Uslaner et al., 2001; Ostrander et al., 2003).

Prior studies showed that the typical and atypical antipsychotics cause increased *c-fos* activity in the striatum and prefrontal cortex, respectively, whereas up-regulation of *c-fos* in the nucleus accumbens was reported for both (Wan et al., 1995; Deutch and Duman, 1996; Verma et al., 2007). In these studies, *c-fos* activity was detected by immunohistochemistry or *in situ* hybridization. These techniques can be limiting due to the sensitivity and specificity of the probes. In addition, they provide limited access to cellular morphology. Since these procedures require the tissue to be fixed, they also severely compromise further biochemical and physiologic investigations.

**Figure 1.**
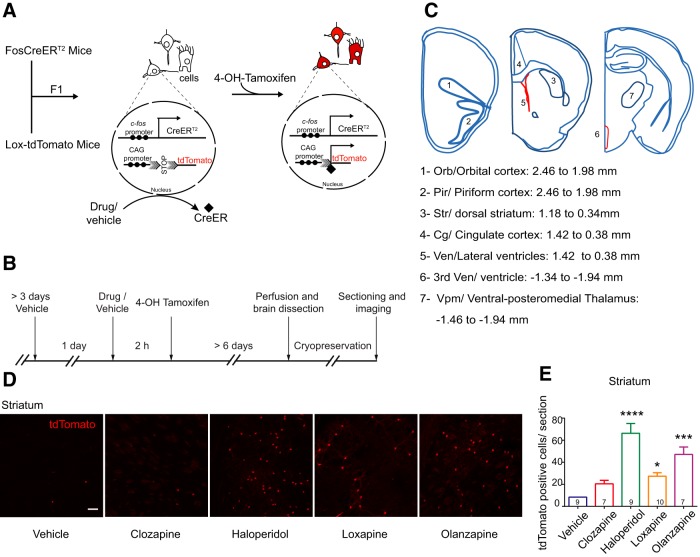
FosTRAP mice showed increased labeling of cells by tdTomato in the striatum on treatment with antipsychotic drugs. ***A***, Schematic representation of the ‘FosTRAP’ system. F1 progeny of FosCreER and Lox-tdTomato mice of the genotype *Fos^CreER/+^R26^AI14/+^* are used. Neural cells that respond (mostly neuronal in morphology; see also Extended Data Fig. 1-1) express CreER^T2^ recombinase, which can only enter the nucleus if bound by Tamoxifen or 4-OH-Tamoxifen. Once CreER^T2^ enters the nucleus it excises the STOP sequence and initiates permanent expression of tdTomato. Promoter region is indicated by solid circles. Arrowheads inside the nucleus indicate initiation and direction of transcription. Solid diamond shape denotes CreER^T2^, shaded arrows represent the LoxP sites. ***B***, Schematic of the experimental protocol. Antipsychotic drugs (at the concentrations indicated) or vehicle were used as the stimulus. 4-OH-Tamoxifen was administered 2 h later. The mice were fixed by perfusion after a minimum of 6 d. The brains were sectioned and imaged. ***C***, Schematic representation of the areas imaged. ***D***, Representative images of the tdTomato labeling induced by various antipsychotics in the dorsolateral striatum. Scale bar: 10 µm. ***E***, Quantification of the number of tdTomato-positive cells per section. Haloperidol showed the maximum number of tdTomato-positive cells in the striatum, followed by Olanzapine and Loxapine. Numbers within the bars represent the number of mice examined in that group. Kruskal–Wallis test was used for statistical significance. Data represented as mean ± SEM; *p < 0.05, ***p < 0.001, ****p < 0.0001.

In this study, we describe the activity induced by antipsychotics, using the ‘FosTrap’ system. This system, designed by Luo and colleagues (Guenthner et al., 2013), permanently marks cells in which *c-fos* is active following the stimulus. TRAP system requires co-administration of Tamoxifen poststimulus, as the *c-fos* promoter drives the expression of the CreER^T2^ recombinase. The presence of the estrogen receptor (ER) binding site allows entry of Cre recombinase into the nucleus only in the presence of Tamoxifen. Therefore, Tamoxifen, coupled with the stimulus, traps “stimulus responsive cells” to permanently express the reporter (Guenthner et al., 2013; Fig. 1*A*). ‘FosTrap’ mice have shown increased *c-fos* activity in specific areas following exposure to a novel context, whisker stimulation, light exposure, etc., which led us to try and identify cells in the brain that are activated by antipsychotics.

10.1523/ENEURO.0220-18.2018.f1-1 Extended Data Figure 1-1 Antipsychotic-induced tdTomato-positive cells had neuronal morphology. ***A–E***, Representative images of the antipsychotic-induced tdTomato-positive cells in various brain regions. The tdTomato-positive cells exhibit neuronal morphology. Scale bar: 10 µm. Download Figure 1-1, EPS file.

We tested two different antipsychotics each from the typical and atypical groups, i.e., Haloperidol, Loxapine, Clozapine, and Olanzapine. Using this system, we screened different cortical and subcortical brain areas for active *c-fos* in response to antipsychotics. In general, we observed that the typical and atypical antipsychotics showed little overlap in the area-wise pattern of activity. Brain areas such as the orbital cortex, piriform cortex, and ventral-posteromedial thalamus (Vpm) were responsive to the atypical drugs while cells in the striatal region responded to typical antipsychotics. 5-HT_2A_ being the major target of Clozapine, we also investigated the role of 5-HT_2A_ in modulating the activity pattern induced by Clozapine using an available 5-HT_2A_ knock-out (KO) mice.

Our results also identified ependymal cells within the ventricles as novel cellular targets of the antipsychotics Clozapine and Olanzapine and heavily modulated by 5-HT_2A_. Briefly, the ‘FosTrap’ system has allowed us to identify live cells in brain regions which are known to be activated or modulated by typical and atypical antipsychotics as well as newer regions and cell types. The system can be used further for potential manipulation and investigation of the “trapped” cells *in vivo* and the circuits they are a part of.

## Materials and Methods

### Animals

Animals were maintained on ad libitum food and water on a 10/14 h light/dark cycle. Experiments were performed during the daytime. Males and females, minimum of eight weeks or older, were used for the experiments. FosCreER mice (c-Fos Cre ERT2 (B6.129(Cg)-*Fos^tm1.1(cre/ERT2)Luo^*/J) and Lox-tdTomato (B6.Cg-*Gt(ROSA)26Sor^tm14(CAG-tdTomato)Hze^*/J) mice were obtained from the Jackson laboratory (stock numbers 021882 and 007914, respectively) and maintained as per The Jackson laboratory guidelines. *Htr2a^-/-^* mice were maintained through heterozygous matings. All animal procedures were performed in accordance with the [National Centre for Biological Sciences] animal care committee’s regulations.

F1 progeny of cross between FosCreER^T2^ mice (*Fos^CreER/+^*) and Lox-tdTomato (*R26^AI14/A14^*) were used for the experiments. The F1 (*Fos^CreER/+^ R26^AI14/+^*) mice were also crossed into *Htr2a^-/-^* background to generate the triple transgenic mice with the genotype *Fos^CreER/+^R26^AI14/+^Htr2a^+/+^*and *Fos^CreER/+^R26^AI14/+^Htr2a^-/-^.* The *Fos^CreER/+^R26^AI14/+^Htr2a^-/-^*were subsequently maintained on the *Htr2a^-/-^* background. Similarly, the *Fos^CreER/+^R26^AI14/+^Htr2a^+/+^*mice were maintained on the *Htr2a^+/+^* background. To rule out the effect of 5-HT_2A_, if any, on maternal rearing, experimental results were verified on a small set of *Htr2a^+/+^* and *Htr2a^-/-^* animals obtained by heterozygous matings of *Fos^CreER/+^ R26^AI14/+^ Htr2a*±.

All the mice were genotyped for the presence of Cre, LoxP, and the *Htr2a* locus.

### Genotyping

Genotyping was performed using polymerase chain reactions with genomic DNA obtained from tail clips.

### Primers

For *Htr2a* KO strain, wild-type (WT) fragment: fragment (1) 408 bp or fragment (2) 200 bp, *Htr2a* mutant fragment: 642 bp.

WT forward (1), CAT GGA AAT TCT CTG TGA AGA CA; WT reverse (1), AGG ATG GTT AAC ATG GAC ACG, WT forward (2), GGT ACC GGT GGC CTT TGC C; WT reverse (2), TAC GGA TAT GGT CCA CAC CGC AAT; mutant forward, AGT TAT TAG GTC CCT CGA AGA GGT; mutant reverse, GGT ACA AGT CCT TGC TGT ACA ATG.

FosCreER mice, WT product: 215, mutant fragment: 293.

Common forward, CAC CAG TGT CTA CCC CTG GA; WT reverse, CGG CTA CAC AAA GCC AAA CT; mutant reverse, CGC GCC TGA AGA TAT AGA AGA.

For Lox-tdTomato mice, WT fragment: 297 bp, mutant fragment: 196 bp.

WT forward, AAG GGA GCT GCA GTG GAG TA; WT reverse, CCG AAA ATC TGT GGG AAG TC; mutant reverse, GGC ATT AAA GCA GCG TAT CC; mutant forward, CTG TTC CTG TAC GGC ATG G.

### Drugs

Clozapine (catalog number 0444) stock: 50 mg/ml, Haloperidol (catalog number 0931) stock: 10 mg/ml, and Olanzapine (catalog number 4349) stock: 50 mg/ml were from Tocris Bioscience and dissolved in DMSO. Loxapine (catalog number L106) stock: 10 mg/ml was from Sigma-Aldrich and dissolved in 0.9% saline. All aqueous solutions were buffered to pH 6–6.5 if required. The drugs were administered intraperitoneally.

4-OH-Tamoxifen (catalog number H6278) was purchased from Sigma-Aldrich. It was prepared as described previously (Guenthner et al., 2013). Briefly, 4-OH-Tamoxifen was dissolved in ethanol at 20 mg/ml and stored at -20°C. 4-OH-Tamoxifen stock in ethanol was mixed with corn oil to achieve the final concentration of 10 mg/ml. The ethanol was evaporated using Centrivap before injection.

### Mouse brain processing

The animals were perfused with 4% paraformaldehyde (PFA) and the brains were dissected out. Brains were postfixed in 4% PFA overnight. Following cryopreservation in 30% sucrose, the brains were sectioned into 40-µm-thick slices.

### Antibody staining

Anti-Vimentin antibody (catalog number ab92547) was from Abcam and the anti-S100β antibody (catalog number Z0311) was from Dako (Agilent Technologies); 0.3% Triton X-100 in 3% milk powder, prepared in PBS, was used for blocking. The sections were incubated with the primary antibody anti-Vimentin (1:300) and anti-S100β (1:500) overnight at 4°C, followed by staining with the secondary antibodies.

### Image acquisition and analysis

The brain slices were imaged on the Olympus FV1000, confocal microscope. Orbital cortex (bregma, 2.46–1.98 mm), Piriform cortex (bregma, 2.46–1.98), Vpm (bregma, -1.46 to -1.94 mm), and the 3rd ventricle (bregma, -1.34 to -1.94 mm) were imaged in every third section. The cingulate cortex (bregma, 1.42–0.38 mm), dorsal striatum (caudate-putamen; bregma, 1.18–0.34 mm) and lateral ventricles (bregma, 1.42–0.38 mm) were examined in every sixth slice. Images were processed using ImageJ 1.47 (NIH) software and the number of cells in each image were counted using Cell profiler (Openware, Broad Institute Imaging Platform). Final data were represented as the number of cells per section averaged from all the slices in the area of interest in both hemispheres. The ependymal cells were often too closely spaced to count individual cells, therefore total intensity was counted per image and the data were plotted as unit intensity/section.

### Statistics

Data are represented as mean ± SEM. Comparison between vehicle and drug treatments were interpreted using one-way ANOVA (or Kruskal–Wallis test where appropriate) with correction for multiple comparisons. *Htr2a^+/+^* and *Htr2a^-/-^* genotypes and treatments were compared using two-way ANOVA with correction for multiple comparisons. Student’s *t* test or Mann–Whitney *U* test was used for comparison between male and female data. The appropriate tests are indicated in the figure legends.

## Results

### Antipsychotics cause the increase in *c-fos* activity in a region-specific manner

To identify cells that respond to antipsychotics by activating *c-fos* we crossed FosCreER^T2^ mice (B6.129(Cg)-*Fos^tm1.1(cre/ERT2)Luo^*/J) to Lox-tdTomato mice (B6.Cg-*Gt(ROSA)26Sor^tm14(CAG-tdTomato)Hze^*/J) and used the F1 progeny (*Fos^CreER/+^ R26^AI14/+^* mice) for the experiments (Fig. 1*A*). The activated cells would express tdTomato, a red fluorescent protein. Following the acute antipsychotic injection, an increase in tdTomato-positive cells was seen in various brain areas. Since the tdTomato is cytoplasmic, the entire cell could be visualized without any staining. The majority of the cells that were labeled also looked neuronal by morphology (Extended Data Fig. 1-1). We have used the term “tdTomato-positive labeling” interchangeably with *c-fos* activation throughout.

As mentioned earlier, previous reports show that typical antipsychotics such as Haloperidol caused activation of *c-fos* in the dorsolateral striatum. Therefore, we analyzed tdTomato labeling in the striatum first to validate our system. Dorsolateral striatum showed the highest number of tdTomato-positive cells on treatment with Haloperidol (vehicle vs Haloperidol, *p* < 0.0001^a^). Loxapine, although considered typical antipsychotic, showed lesser numbers of activated cells in the striatum compared to Haloperidol (vehicle vs Loxapine, *p* = 0.0407^a^; Fig. 1*D*,*E*). Interestingly, Olanzapine which belongs to the atypical class of antipsychotics showed a stronger *c-fos* response in the striatum (vehicle vs Olanzapine, *p* = 0.0003^a^; 5.5-fold over control) compared to its structural analog Clozapine but lesser than haloperidol (Fig. 1*D*,*E*). This was reproducibly observed suggesting that the labeling process was robust and antipsychotic specific.

In conclusion, the TRAP system could successfully and permanently label cells within specific brain regions in a specific antipsychotic-dependent manner. The area in which these cells were located was similar to what was expected of the typical antipsychotics based on previous literature.

### Cortical and thalamic subregions are more responsive to Clozapine and Olanzapine

**Figure 2.**
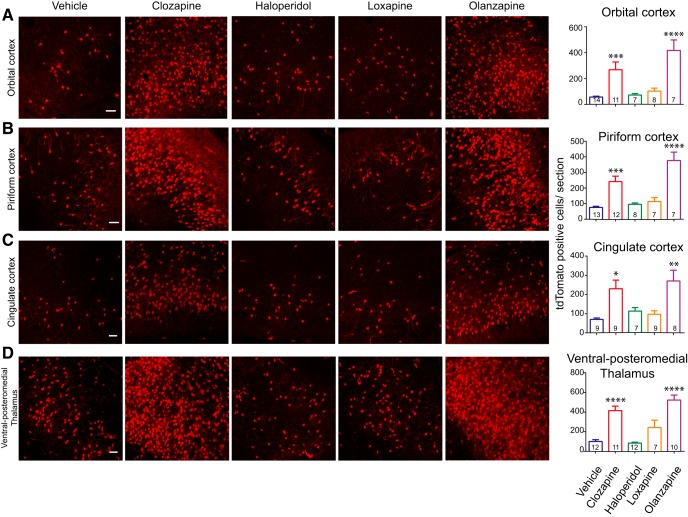
Clozapine and Olanzapine showed increased *c-fos* activity in various cortical regions and Vpm. ***A–D***, Representative images of the tdTomato-labeled cells in the orbital cortex, piriform cortex, cingulate cortex, and Vpm. Scale bar: 10 µm. The graphs on the right show quantification of the number of tdTomato-positive cells per section. In all of these regions, Clozapine and Olanzapine significantly increased the number of tdTomato-positive cells compared to vehicle. The number of cells induced by Haloperidol or Loxapine was comparable to that of the vehicle. Numbers in the bars represent the number of mice examined in that group. One-way ANOVA or Kruskal–Wallis test was used. Data represented as mean ± SEM; *p < 0.05, **p < 0.01, ***p < 0.001, ****p < 0.0001. See also Extended Data Figure 2-1.

While Clozapine labeled the least number of cells in the striatum, certain cortical regions (Fig. 1*E*) were very responsive to Clozapine. Clozapine and Olanzapine caused *c-fos* activity patterns similar to each other in the cortical structures that we examined, unlike the striatum (Figs. 1*B*, 2*A–C*). These atypical antipsychotics caused significantly higher increases in tdTomato-positive cells in the orbital cortex (vehicle vs Clozapine, *p* = 0.0006; vehicle vs Olanzapine, *p* < 0.0001^a^), piriform cortex (vehicle vs Clozapine, *p* = 0.0002; vehicle vs Olanzapine, *p* < 0.0001^a^), and anterior cingulate cortex (vehicle vs Clozapine, *p* = 0.01; vehicle vs Olanzapine, *p* = 0.0027^a^), compared to the vehicle. In these brain areas, the number of tdTomato-positive cells induced by Haloperidol and Loxapine was comparable to the vehicle.

10.1523/ENEURO.0220-18.2018.f2-1 Extended Data Figure 2-1 Gender dependence and dose dependence of Clozapine-induced *c-fos* activity. ***A***, ***B***, The graphs show quantification of the tdTomato-positive labelling in various brain regions following (***A***) Clozapine treatment and (***B***) Vehicle treatment. There were no significant differences between the male and female mice for any of the brain regions tested. Minimum of four mice was analyzed per gender per brain region. Clozapine-treated striatum group had three male mice. Student’s *t* test or Mann–Whitney *U* test was performed to compare male and female data. Data represented as mean ± SEM; *p < 0.05, **p < 0.01, ***p < 0.001, ****p < 0.0001. ***C–F***, Quantification of the dose-response curve to Clozapine administered at 0, 5, and 20 mg/kg is shown. The orbital cortex, piriform cortex, and cingulate cortex showed slight increases in tdTomato labelling at 5 mg/kg, which reached significance at 20 mg/kg. Vpm showed significantly more tdTomato-positive labelling at 5 and 20 mg/kg. Numbers within the bars represent the number of mice examined in that group. One-way ANOVA or Kruskal–Wallis test, as appropriate, was used. Data represented as mean ± SEM; *p < 0.05, **p < 0.01, ***p < 0.001, ****p < 0.0001. Download Figure 2-1, EPS file.

Previous studies have reported higher Clozapine-induced *c-fos* activity in the cortical regions (Deutch and Duman, 1996; Ohashi et al., 2000; Verma et al., 2007); however, *c-fos* activity in the orbital cortex and piriform cortex had not been reported.

Along with the cortical regions, *c-fos* activity was also noticeable in the Vpm (Figs. 1*B*, 2*D*). Clozapine and Olanzapine showed higher numbers of tdTomato-positive cells in the Vpm compared to vehicle (vehicle vs Clozapine, *p* < 0.0001; vehicle vs Olanzapine, *p* < 0.0001^a^). Vpm is a part of thalamus which relays oral and facial sensory information to the somatosensory cortex. There are a few reports of antipsychotic-induced *c-fos* activity in the thalamus (Cohen, 1995; Deutch et al., 1995; Rajkumar et al., 2013), although antipsychotic-induced *c-fos* responses have not been reported in Vpm.

Most of the behavioral or biochemical studies have used only male mice, and the responses of female mice remain largely undetermined. Therefore, in this study, we also analyzed the antipsychotic-induced *c-fos* responses in male as well as female mice. We systematically compared *c-fos* activity in various brain regions between males and females. We did not observe any gender-specific difference in any of the regions tested (Extended Data Fig. 2-1*A*,*B*) with either Clozapine or vehicle treatment. Hence, females were also included in the subsequent experiments and analysis.

Since antipsychotic effects are dose dependent, we analyzed the dose response to Clozapine. Previously, significant differences in Clozapine-induced sedation between *Htr2a^+/+^* and *Htr2a^-/-^* mice have been reported at 5 mg/kg (McOmish et al., 2012; Joshi et al., 2017), whereas doses of Clozapine around 20 mg/kg have been used very extensively to assess Clozapine-induced *c-fos* responses (Wan et al., 1995; Deutch and Duman, 1996; Badiani et al., 1999). Therefore, we tested 0, 5, and 20 mg/kg of Clozapine. At 5 mg/kg of Clozapine, most brain regions showed a trend toward increased *c-fos* activity (Extended Data Fig. 2-1*C*,*F*). However, at the higher dose of 20 mg/kg, Clozapine significantly increased the number of tdTomato-positive cells in the cortical regions. Doses of the other antipsychotics that we tested were chosen based on the previous literature on behavioral and biochemical effects of these drugs (Deutch and Duman, 1996; Robertson and Fibiger, 1996; Cope et al., 2005; orbital cortex: vehicle vs Clozapine 20 mg/kg, *p* = 0.0005; piriform cortex: vehicle vs Clozapine 20 mg/kg, *p* < 0.0001; cingulate cortex: vehicle vs Clozapine 20 mg/kg, *p* = 0.0130; Vpm: vehicle vs Clozapine 5 mg/kg, *p* = 0.0147, vehicle vs Clozapine 20 mg/kg, *p* < 0.0001^a^).

### Ependymal cells are a novel cellular target of Clozapine and Olanzapine

**Figure 3.**
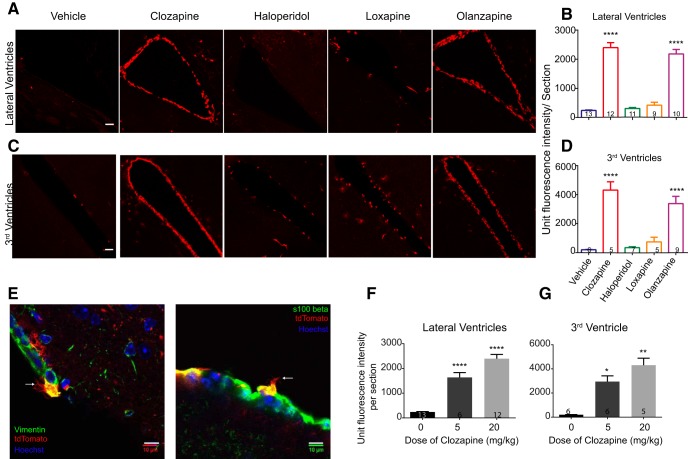
Ependymal cells were a novel cellular target of Clozapine and Olanzapine. ***A***, ***C***, Representative images of the tdTomato-positive labeling along the lateral ventricles and the 3rd ventricle, on treatment with various antipsychotics or vehicle. Scale bar: 10 µm. tdTomato fluorescence within cells were seen on treatment with Clozapine and Olanzapine. ***B***, ***D***, Quantification of the tdTomato fluorescence in cells lining the ventricles. Clozapine and Olanzapine showed significantly more fluorescence compared to vehicle. Haloperidol and Loxapine-induced *c-fos* activity along the ventricles were not significantly different from that of the vehicle. Numbers in the bars represent the number of mice examined in the group. One-way ANOVA or Kruskal–Wallis test, as appropriate, was used. ***E***, tdTomato-positive cells lining the ventricles showed multiple cilia and stained positive for markers of ependymal cells (see also Extended Data Fig. 3-1). White arrows point toward ciliated structures. Scale bar: 10 µm. ***F***, ***G***, Graphs represent dose response to Clozapine. Cells lining the ventricles showed significantly higher tdTomato-positive labeling even at 5 mg/kg dose of Clozapine. Numbers in the bars represent the number of mice examined. One-way ANOVA or Kruskal–Wallis test, as appropriate, was used. Data represented as mean ± SEM; *p < 0.05, **p < 0.01, ****p < 0.0001.

Among the areas that showed *c-fos* expression with atypical antipsychotics were the ventricular regions. We observed consistent tdTomato labeling along the ventricles in Clozapine and Olanzapine-treated animals. This labeling was observed in the lateral ventricles as well as the 3rd ventricle (Fig. 3*A–D*) and the 4th ventricle (data not shown; lateral ventricles: vehicle vs Clozapine *p* < 0.0001, vehicle vs Olanzapine *p* < 0.0001; 3rd ventricles: vehicle vs Clozapine *p* < 0.0001, vehicle vs Olanzapine *p* < 0.0001^a^). Haloperidol and Loxapine showed very little labeling along the ventricles (Fig. 3*A–D*). Curiously, the cells lining the ventricles were more responsive to lower doses of Clozapine than the cortical regions and showed significantly higher responses even at 5 mg/kg of Clozapine (Fig. 3*F*,*G*; lateral ventricle: vehicle vs Clozapine 5 mg/kg *p* < 0.0001, vehicle vs Olanzapine *p* < 0.0001; 3rd ventricle: vehicle vs Clozapine 5 mg/kg *p* = 0.0.0328, vehicle vs Olanzapine *p* = 0.0015^a^).

10.1523/ENEURO.0220-18.2018.f3-1 Extended Data Figure 3-1 Antipsychotic-induced tdTomato-positive cells along the ventricles had multiple cilia. ***A–D***, Representative images of the tdTomato-positive cells along the ventricles under high magnification. The tdTomato protein filled the entire cell volume and the cilia which were identifiable (white arrows) at higher magnification. Scale bar: 10 µm. Download Figure 3-1, EPS file.

Atypical antipsychotics have been shown to stimulate neurogenesis in the subventricular zone, the subgranular zone in the hippocampus and in the cortex (Wakade et al., 2002; Halim et al., 2004; Kodama et al., 2004; Wang et al., 2004). Therefore, we considered the possibility that the tdTomato-positive cells lining the ventricles could be the neural stem cells. However, under high magnification these cells showed multiple cilia (Extended Data Fig. 3-1; Fig. 3*E*), suggesting that these were ependymal cells (Brightman and Palay, 1963; Del Bigio, 1995; Jiménez et al., 2014). Additionally, these cells stained positive for the ependymal cell marker Vimentin and S100β (Fig. 3*E*; Didier et al., 1986; Bruni, 1998; Schnitzer et al., 1981). Therefore, we conclude that these were ependymal cells and are novel cellular targets of the atypical antipsychotics that we tested.

### The absence of 5-HT_2A_ does not alter the number of tdTomato-positive cells in the cortical and thalamic region

5-HT_2A_ has been considered an important target for the therapeutic efficacy of Clozapine seen in mouse models of schizophrenia (Fribourg et al., 2011; Schmid et al., 2014; Moreno et al., 2016). 5-HT_2A_ expression has been reported in the various regions of cortex such as piriform cortex, orbital cortex, cingulate cortex, etc. (Xu and Pandey, 2000; Miner et al., 2003). 5-HT_2A_ levels are also reported to increase in postmortem brain (cortical) samples of drug naïve schizophrenic patients (González-Maeso et al., 2008; Muguruza et al., 2013) and chronic treatment with Clozapine reduces levels of 5-HT_2A_ in patients and animal models (Yadav et al., 2011b; Muguruza et al., 2013). Therefore, we examined whether the 5-HT_2A_ receptor would modulate the observed pattern of *c-fos* activity with Clozapine. We crossed *Htr2a^-/-^* mouse strain (obtained from Joshi et al., 2017) into the FosCreER^T2^ (c-Fos Cre ERT2 (B6.129(Cg)-*Fos^tm1.1(cre/ERT2)Luo^*/J) and Lox-tdTomato (B6.Cg-*Gt(ROSA)26Sor^tm14(CAG-tdTomato)Hze^*/J) background.

**Figure 4.**
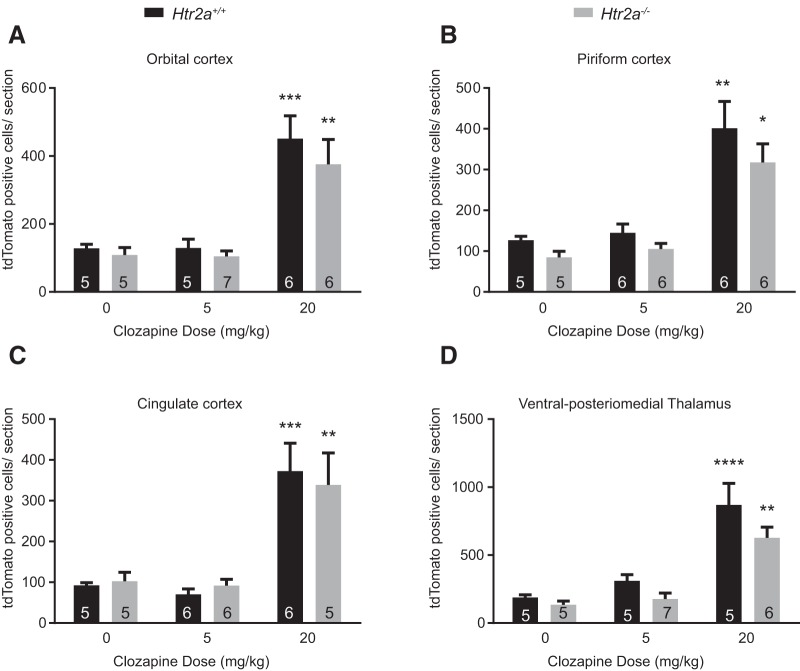
Clozapine-induced *c-fos* activity in the cortical regions and Vpm was largely intact in the *Htr2a^-/-^* mice. ***A–D***, *Htr2a^+/+^* and *Htr2a^-/-^* mice were not different in the number of *c-fos*-positive cells in the orbital cortex, cingulate cortex, piriform cortex, and Vpm, at 5 or 20 mg/kg. Numbers in the bars represent the number of mice examined in that group. Two-way ANOVA was used. Data are represented as mean ± SEM. * comparison between vehicle and treatment for the same genotype. # represents the comparison between WT and mutant under the same conditions; *p < 0.05, **p < 0.01, ***p < 0.001, ****p < 0.0001.

We observed that Clozapine-induced *c-fos* activity in the cortical and thalamic regions was largely unaffected in the *Htr2a^-/-^* background, at both the doses tested, 5 and 20 mg/kg (Fig. 4*A–D*). This suggested that 5-HT_2A_ had minimal or no role in determining the number of tdTomato-positive neurons in response to Clozapine, in these regions. This result was surprising especially considering that these cortical areas are known to express 5-HT_2A_. This would suggest that the *c-fos* activity resulting in tdTomato-positive cells in the cortex arise from the complex circuitry and are not derived from direct interactions between 5-HT_2A_ and Clozapine (orbital cortex: WT, vehicle vs Clozapine 20 mg/kg, *p* = 0.0005; KO, vehicle vs Clozapine 20 mg/kg, *p* = 0.0055; piriform cortex: WT, vehicle vs Clozapine 20 mg/kg, *p* = 0.0003; KO, vehicle vs Clozapine 20 mg/kg, *p* = 0.0028; cingulate cortex: WT, vehicle vs Clozapine 20 mg/kg, *p* = 0.0023; KO, vehicle vs Clozapine 20 mg/kg, *p* = 0.0210; Vpm; WT, vehicle vs Clozapine 20 mg/kg, *p* < 0.0001; KO, vehicle vs Clozapine 20 mg/kg, *p* = 0.0210^a^).

### Clozapine-induced tdTomato labeling in the ependymal cells is modulated by 5-HT_2A_

**Figure 5.**
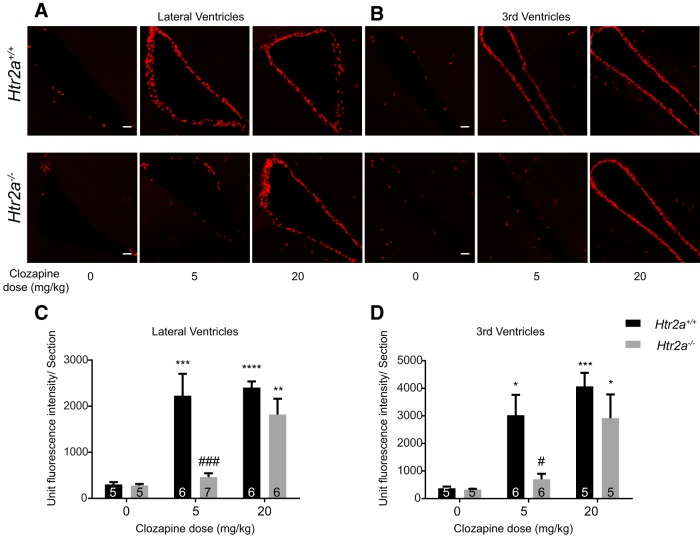
Clozapine-induced *c-fos* activity in the ependymal cells was diminished by deletion of 5-HT_2A_. ***A***, ***B***, Representative images of the lateral ventricles and the 3rd ventricles. tdTomato labeling was strikingly diminished in the *Htr2a^-/-^* mice at 5 mg/kg of Clozapine. However, at 20 mg/kg of Clozapine increased levels of activity were seen. Scale bar: 10 µm. ***C***, ***D***, The lateral and the 3rd ventricles showed reduced tdTomato fluorescence at 5 mg/kg in the *Htr2a^-/-^* mice compared to the *Htr2a^+/+^*. Fluorescence levels were not statistically different between the *Htr2a^+/+^* and *Htr2a^-/-^* mice at 20 mg/kg of Clozapine. Numbers in the bars represent the number of mice assayed in that group. Two-way ANOVA was used for statistical significance. Data are represented as mean ± SEM. * comparison between vehicle and treatment for the same genotype. # represents the comparison between WT and mutant under the same conditions; *p < 0.05, **p < 0.01, ***p < 0.001, ****p < 0.0001.

Unlike the rest of the brain regions, Clozapine-induced tdTomato labeling of ependymal cells was dramatically diminished in the *Htr2a^-/-^* mice even at 5 mg/kg of Clozapine (Fig. 5*A*,*B*). On increasing the dose to 20 mg/kg, labeling of the ependymal cells was observed in the *Htr2a^-/-^* mice and was statistically indistinguishable from the numbers observed in *Htr2a^+/+^* mice (Fig. 5*C*,*D*). These data suggest that the genetic deletion of 5-HT_2A_ receptor modulates the Clozapine-induced *c-fos* activity in the ependymal cells, even at low doses of Clozapine (lateral ventricle: WT, vehicle vs Clozapine 5 mg/kg, *p* = 0.0003, vehicle vs Clozapine 20 mg/kg, *p* < 0.0001; KO, vehicle vs Clozapine 20 mg/kg, *p* = 0.0043, WT vs KO at Clozapine 5 mg/kg, *p* = 0.0003; 3rd ventricle; WT, vehicle vs Clozapine 5 mg/kg, *p* = 0.0134, vehicle vs Clozapine 20 mg/kg, *p* = 0.0006; KO, vehicle vs Clozapine 20 mg/kg, *p* = 0.0226; WT vs KO at Clozapine 5 mg/kg, *p* = 0.0270^a^).

In summary, the FosTrap system has led to the identification of brain areas such as orbital cortex, piriform cortex, Vpm, etc., as areas where *c-fos* gets consistently and reproducibly induced by the antipsychotics, Clozapine and Olanzapine. The labeled cells are neuronal and can be potentially accessed “live.” Furthermore, we observed, with some surprise, that absence 5-HT_2A_ did not alter the number of tdTomato-positive cells at low or high doses of atypical antipsychotics in cortical or thalamic regions that were tested. However, a clear and dose-dependent effect on the tdTomato labeling of ependymal cells was seen with these drugs. To the best of our knowledge, this is the first report of the effect of antipsychotics on ependymal cells.

## Discussion

A thorough understanding of the mechanism of existing antipsychotics holds the key to develop safer and effective drugs. The pharmacology of the available antipsychotics has been studied quite well, although the neural correlates of these need more investigation. In this study, we have addressed a part of this issue and opened a new approach using the FosTrap system devised by Luo and colleagues (Guenthner et al., 2013).

Our experiments have already provided novel information as well as corroborated some of the available results. Haloperidol-induced *c-fos* activity in the striatum has been reported before. This activity has also been attributed to its D2 antagonism and the cataleptic side effect induced by Haloperidol (Kapur et al., 1995, 2000; Wadenberg et al., 2000). In addition to the typical antipsychotics that we tested (Haloperidol and Loxapine), Olanzapine also showed induction of *c-fos* in the striatum. This was a surprising finding owing to the atypical classification of Olanzapine. However, some studies have reported catalepsy induced by Olanzapine in animal models (Kalkman et al., 1997; Ahlqvist et al., 2003).

A parsimonious and widely-accepted explanation for the pattern of antipsychotic-induced activity in the striatum (Fig. 1*D*) is based on the relative affinities of these drugs to the receptors. As mentioned above, D2 antagonism is thought to promote catalepsy, whereas 5-HT_2A_ antagonism is thought to improve catalepsy-like behavior (Ansah et al., 2011; Creed-Carson et al., 2011). The balance of affinities for D2 and 5-HT_2A_ may indeed determine the *c-fos* activity in the striatum. Among the four antipsychotics tested, Haloperidol has the highest affinity for D2 and Clozapine the least. Olanzapine and Loxapine have 10-fold more affinity for D2 than Clozapine and more affinity for 5-HT_2A_ than Haloperidol (Roth et al., 2004).

Clozapine and Olanzapine-induced increases in *c-fos* activity in the cortical areas have useful implications, particularly because these regions have been associated with the pathophysiology of schizophrenia and other mental disorders. For example, reduction in gray matter in the anterior cingulate cortex and orbitofrontal cortex has been observed in postmortem samples of schizophrenia patients (Pantelis et al., 2003; Fornito et al., 2009). Reduction in the volume of olfactory bulb and deficits in the olfactory capacity have also been reported (Moberg et al., 1999; Turetsky et al., 2000). Moreover, these cortical areas have been associated with cognitive functions such as reward learning, decision-making (Rushworth et al., 2011), working memory (Barbey et al., 2011), etc., *c-fos* activity in the piriform cortex has also been associated with antidepressant effects (Sibille et al., 1997). Therefore, *c-fos* induction in these brain regions may indeed correlate with the effect of antipsychotics on the negative symptoms and the cognitive symptoms of schizophrenia.

Antipsychotic-induced increased *c-fos* activity in the Vpm is a novel finding. Hallucinogens such as LSD and DOI, 5-HT_2A_ agonists, have been shown to bring about hallucinogen-specific gene regulation in the somatosensory cortex (González-Maeso et al., 2003). Therefore, the increased *c-fos* activity in the Vpm, in response to Clozapine and Olanzapine, may be related to the circuitry underlying modulation by antipsychotics in the somatosensory cortex.

Certain behavioral effects of atypical antipsychotics, for example, sedation, are shown to be dependent on 5-HT_2A_ (McOmish et al., 2012; Williams et al., 2012; Joshi et al., 2017), while other biochemical effects are reported to be independent of 5-HT_2A_ (Bortolozzi et al., 2010; Yadav et al., 2011a). Taking into account the high affinity of Clozapine for 5-HT_2A_ and receptor’s expression in the cortex, we expected Clozapine to show a significant decrease in the number of tdTomato-positive cells in the cortical areas in *Htr2a^-/-^* mice. Surprisingly, the response of Clozapine, in the number of tdTomato-positive cells, was statistically indistinguishable in the cortical areas and the Vpm, between the *Htr2a* ^-/-^ and *Htr2a^+/+^* backgrounds. This suggests that the increase in *c-fos* activity in the cortical areas due to atypical antipsychotics is 5-HT_2A_ independent and perhaps likely due to the interaction of the antipsychotics with other receptors. It would certainly be of interest to determine the strength of the response and any variation in the transcriptome of the “tdTomato-positive cells” in the absence of 5-HT_2A_; however, it is beyond the scope of the current report. The strength of the response in the TRAP system is limited to the number of cells activated, and it is not designed to report on the levels of c-Fos in the cell.

In our system, we saw c*-fos* activity predominantly in cells with neuronal morphology, except in case of the ependymal cells. Antipsychotic-induced activation of the ependymal cells is an important finding from this study. This finding is novel and particularly intriguing because of the functional properties of this multiciliated cell type. Ependymal cells play crucial role in cortical development (Jiménez et al., 2014). Ependymal cells form a barrier between CSF and brain tissue and are involved in diverse functions such as the production and circulation of CSF (Nelson and Wright, 1974; Nielsen et al., 1997; Tissir et al., 2010) and transport of water molecules (Nielsen et al., 1997; Jiménez et al., 2014). Ciliopathies are associated with mental retardation and neurodevelopmental disorders (Lee and Gleeson, 2011). Moreover, several genes underlying neuropsychiatric disorders, have been found to be localized to or involved in the formation and maintenance of primary cilia, for example, schizophrenia-associated gene DISC1 (Marley and von Zastrow, 2010, 2012). *c-fos* activity in ependymal cells has been reported in response to formalin-induced acute pain (Palkovits et al., 2007), posthypoxia seizures (Gunn et al., 1990), and on treatment with the antidepressants such as Rolipram (Dragunow and Faull, 1989). Importantly, ependymal cells also provide a niche for neural stem cells (Lim et al., 2000; Tramontin et al., 2003; Luo et al., 2008). Thus, our results are particularly intriguing in light of the role of the “5-HT_2A_ expressing” Paneth cells in providing the niche for intestinal stem cells (Sato et al., 2011).

In Clozapine-induced dose response, most brain areas did not show significant *c-fos* activity at the lower dose of 5 mg/kg. However, strikingly large number of ependymal cells showed response even at the lowest dose of Clozapine, i.e., 5 mg/kg. This would indicate that ependymal cells are more sensitive to Clozapine than cells in other brain areas that we examined.

Additionally, serotonin has been shown to increase ciliary beat frequency, which can be blocked by the broad spectrum 5-HT_2_ receptor antagonist, mianserin (Nguyen et al., 2001). Therefore, the interaction of antipsychotics with the ependymal cells may regulate ciliary beat frequency and in turn the flow of CSF and transport of molecules through CSF. Also, it is important to note that we cannot exclude the involvement of the activated ependymal cells in the side effects of antipsychotics rather than the therapeutic effects.

Clozapine-induced *c-fos* activity in the ependymal cells was absent in the *Htr2a^-/-^* mice at 5 mg/kg. Interestingly, *c-fos* activity is regained when the dose of Clozapine is increased to 20 mg/kg, suggesting that 5-HT_2A_ facilitates the activation of ependymal cells in response to Clozapine at the lower doses and the *Htr2a^-/-^* mice should serve to ascertain other pathways that may be also involved when Clozapine is present at higher concentrations.

“5-HT_2A_ independent” pathway of antipsychotic-induced activation of the ependymal cell would be of interest to pursue. Most antipsychotics, have a broad spectrum of GPCR targets (Roth et al., 2004). Therefore, systematic comparison of the differences in receptor binding profile of the four antipsychotics that we tested, can reveal interesting candidates such as the histamine receptor H1 (Roth et al., 2004). Incidentally, histamine has been shown to increase c-Fos immunoreactivity in the ependymal cells (Palkovits et al., 2007). Dopamine receptors and other 5-HT receptors would also be the other candidates and interestingly enough, 5-HT6 receptor and dopamine receptors (D1, D2, and D5) are reported to localize to primary cilia (Marley and von Zastrow, 2010; Hu et al., 2017). One has to also keep “functional selectivity” in mind, i.e., when GPCRs interact with different antipsychotics (Yadav et al., 2011b; Raote et al., 2013). Importantly, the 5-HT_2A_-independent pathway of activation of ependymal cells could be GPCR independent and be regulated by the other intracellular targets of antipsychotics instead (Yadav et al., 2011a).

It is also important to keep certain limitations of this study in mind. Firstly, we have used WT or *Htr2a^-/-^* mice, which by themselves are clearly not a model for psychosis. We have so far only looked at the acute effects of the antipsychotic treatment. Antipsychotic treatments in patients are typically chronic and therapeutic effects often begin to appear later in the treatment, whereas some side effects do appear in the early part of the treatment. Therefore, it is important to compare both the acute and chronic patterns of antipsychotic activity. The patterns of activity observed by us may also be limited by the differences in the efficiency of “TRAP” in different neural subtypes or brain regions. Variation in efficiencies can in part address the lack of antipsychotic-induced *c-fos* activity in the nucleus accumbens and medial prefrontal cortex, which have been reported as the targets of antipsychotics earlier.

Nonetheless, the TRAP system has allowed us to re-examine antipsychotic-induced patterns of activity in the brain and led to the identification of novel targets. Trap system provides distinct advantages over the existing methods: (1) better visualization of cellular morphology and potential connectivity; (2) permanent marking/labeling of the cells; and (3) killing of the animals immediately after the stimulus is not required.

This approach can also be extended with the optogenetic and biochemical techniques to shed light on the physiologic role of various cell types and brain areas in the functioning of antipsychotic drugs in detail and also can be extended to other genes.[Table T1]

**Table 1: T1:** Table of statistics

Figure	Data structure	Type of test		Confidence interval	Upper	Lower	Mean ranks
(1*E*, Cpu	Not normal	Kruskal– Wallis test		Vehicle			5
				Clozapine			16.86
				Haloperidol			34.78
				Loxapine			21.2
				Olanzapine			30.71
2*A*, Orb	Not normal	Kruskal– Wallis test		Vehicle			14.14
				Clozapine			38.18
				Haloperidol			19.57
				Loxapine			22.13
				Olanzapine			45.57
2*B*, Pir	Normal	One-way ANOVA		Vehicle - Clozapine 20 mg/kg	-71.0245	-261.449	
				Vehicle - Haloperidol 1 mg/kg	86.82508	-126.926	
				Vehicle - Loxapine 1.5 mg/kg	73.44772	-149.555	
				Vehicle - Olanzapine 16 mg/kg	-192.978	-415.981	
				Vehicle - Clozapine 5 mg/kg	53.0639	-181.707	
2*C*, Cg	Not normal	Kruskal– Wallis test		Vehicle - Clozapine 20 mg/kg	-33.2025	-307.102	
				Vehicle - Haloperidol 1 mg/kg	103.291	-189.52	
				Vehicle - Loxapine 1.5 mg/kg	110.014	-163.885	
				Vehicle - Olanzapine 16 mg/kg	-59.3778	-341.707	
				Vehicle - Clozapine 5 mg/kg	40.4602	-265.769	
2*D*, Vpm	Normal	One-way ANOVA		Vehicle - Clozapine 20 mg/kg	-147.234	-436.742	
				Vehicle - Haloperidol 1 mg/kg	162.7995	-120.346	
				Vehicle - Loxapine 1.5 mg/kg	22.91145	-306.942	
				Vehicle - Olanzapine 16 mg/kg	-271.852	-568.817	
				Vehicle - Clozapine 5 mg/kg	-1.71831	-331.572	
3*B*, lateral ven	Not normal	Kruskal– Wallis test		Vehicle			13.62
				Clozapine			45.58
				Haloperidol			18.91
				Loxapine			19.56
				Olanzapine			43.2
3*D*, 3rd ven	Normal	One-way ANOVA		Control - Clozapine 20 mg/kg	-2882.17	-5970.32	
				Control - Haloperidol 1 mg/kg	1244.376	-1592.95	
				Control - Loxapine 1.5 mg/kg	1004.714	-2083.43	
				Control - Olanzapine 16 mg/kg	-1947.88	-4635.77	
				Control - Clozapine 5 mg/kg	-1272.19	-4216.62	
3*F*, dose response, lateral ven	Normal	One-way ANOVA		Vehicle - Clozapine 5 mg/kg	-906.076	-1881.76	
				Vehicle - Clozapine 20 mg/kg	-1754.7	-2546.08	
3*G*, dose response, 3rd ven	Not normal	Kruskal– Wallis test		Vehicle			3.5
				Clozapine 5 mg/kg			10.5
				Clozapine 20 mg/kg			13.8
4*A*, Orb	Normal	Two-way ANOVA		Clozapine 0 mg/kg:Htr2a*^*+/+*^* - Clozapine 0 mg/kg:Htr*2a^*-/-*^*	239.4678	-200.334	
				Clozapine 0 mg/kg:Htr2a*^*+/+*^* - Clozapine 5 mg/kg:Htr2a*^*+/+*^*	218.9879	-220.814	
				Clozapine 0 mg/kg:Htr2a*^*+/+*^* - Clozapine 20 mg/kg:Htr2a*^*+/+*^*	-112.395	-533.474	
				Clozapine 0 mg/kg:Htr*2a^*-/-*^* - Clozapine 5 mg/kg:Htr*2a^*-/-*^*	207.5292	-199.648	
				Clozapine 0 mg/kg:Htr*2a^*-/-*^* - Clozapine 20 mg/kg:Htr*2a^*-/-*^*	-56.0882	-477.167	
				Clozapine 5 mg/kg:Htr2a*^*+/+*^* - Clozapine 5 mg/kg:Htr*2a^*-/-*^*	228.0091	-179.169	
				Clozapine 20 mg/kg:Htr2a*^*+/+*^* - Clozapine 20 mg/kg:Htr*2a^*-/-*^*	276.6153	-124.867	
4*B*, pir	Normal	Two-way ANOVA		Clozapine 0 mg/kg:Htr2a*^*+/+*^* - Clozapine 0 mg/kg:Htr*2a^*-/-*^*	222.2253	-138.417	
				Clozapine 0 mg/kg:Htr2a*^*+/+*^* - Clozapine 5 mg/kg:Htr2a*^*+/+*^*	162.3596	-198.282	
				Clozapine 0 mg/kg:Htr2a*^*+/+*^* - Clozapine 20 mg/kg:Htr2a*^*+/+*^*	-102.107	-447.395	
				Clozapine 0 mg/kg:Htr*2a^*-/-*^* - Clozapine 5 mg/kg:Htr*2a^*-/-*^*	152.3912	-192.897	
				Clozapine 0 mg/kg:Htr*2a^*-/-*^* - Clozapine 20 mg/kg:Htr*2a^*-/-*^*	-60.0426	-405.331	
				Clozapine 5 mg/kg:Htr2a*^*+/+*^* - Clozapine 5 mg/kg:Htr*2a^*-/-*^*	212.2569	-133.031	
				Clozapine 20 mg/kg:Htr2a*^*+/+*^* - Clozapine 20 mg/kg:Htr*2a^*-/-*^*	248.5787	-80.6407	
4*C*, cg	Normal	Two-way ANOVA		Clozapine 0 mg/kg:Htr2a*^*+/+*^* - Clozapine 0 mg/kg:Htr*2a^*-/-*^*	202.757	-223.344	
				Clozapine 0 mg/kg:Htr2a*^*+/+*^* - Clozapine 5 mg/kg:Htr2a*^*+/+*^*	235.1579	-190.943	
				Clozapine 0 mg/kg:Htr2a*^*+/+*^* - Clozapine 20 mg/kg:Htr2a*^*+/+*^*	-76.2599	-484.22	
				Clozapine 0 mg/kg:Htr*2a^*-/-*^* - Clozapine 5 mg/kg:Htr*2a^*-/-*^*	214.9152	-193.045	
				Clozapine 0 mg/kg:Htr*2a^*-/-*^* - Clozapine 20 mg/kg:Htr*2a^*-/-*^*	-23.1811	-449.282	
				Clozapine 5 mg/kg:Htr2a*^*+/+*^* - Clozapine 5 mg/kg:Htr*2a^*-/-*^*	182.5143	-225.446	
				Clozapine 20 mg/kg:Htr2a*^*+/+*^* - Clozapine 20 mg/kg:Htr*2a^*-/-*^*	237.6954	-170.265	
4*D*, Vpm	Normal	Two-way ANOVA		Clozapine 0 mg/kg:Htr2a*^*+/+*^* - Clozapine 0 mg/kg:Htr*2a^*-/-*^*	410.9157	-303.868	
				Clozapine 0 mg/kg:Htr2a*^*+/+*^* - Clozapine 5 mg/kg:Htr2a*^*+/+*^*	235.4589	-479.325	
				Clozapine 0 mg/kg:Htr2a*^*+/+*^* - Clozapine 20 mg/kg:Htr2a*^*+/+*^*	-323.801	-1038.59	
				Clozapine 0 mg/kg:Htr*2a^*-/-*^* - Clozapine 5 mg/kg:Htr*2a^*-/-*^*	289.2806	-372.481	
				Clozapine 0 mg/kg:Htr*2a^*-/-*^* - Clozapine 20 mg/kg:Htr*2a^*-/-*^*	-149.546	-833.899	
				Clozapine 5 mg/kg:Htr2a*^*+/+*^* - Clozapine 5 mg/kg:Htr*2a^*-/-*^*	464.7374	-197.024	
				Clozapine 20 mg/kg:Htr2a*^*+/+*^* - Clozapine 20 mg/kg:Htr*2a^*-/-*^*	585.1711	-99.1824	

5*C*, Ven	Normal	Two-way ANOVA		Clozapine 0 mg/kg:Htr2a*^*+/+*^* - Clozapine 0 mg/kg:Htr*2a^*-/-*^*	1303.436	-1218.51	
				Clozapine 0 mg/kg:Htr2a*^*+/+*^* - Clozapine 5 mg/kg:Htr2a*^*+/+*^*	-718.805	-3133.38	
				Clozapine 0 mg/kg:Htr2a*^*+/+*^* - Clozapine 20 mg/kg:Htr2a*^*+/+*^*	-894.704	-3309.28	
				Clozapine 0 mg/kg:Htr*2a^*-/-*^* - Clozapine 5 mg/kg:Htr*2a^*-/-*^*	968.4059	-1366.46	
				Clozapine 0 mg/kg:Htr*2a^*-/-*^* - Clozapine 20 mg/kg:Htr*2a^*-/-*^*	-352.59	-2767.17	
				Clozapine 5 mg/kg:Htr2a*^*+/+*^* - Clozapine 5 mg/kg:Htr*2a^*-/-*^*	2878.763	660.2965	
				Clozapine 20 mg/kg:Htr2a*^*+/+*^* - Clozapine 20 mg/kg:Htr*2a^*-/-*^*	1735.683	-566.528	
5*D*, 3rd Ven	Normal	Two-way ANOVA		Clozapine 0 mg/kg:Htr2a*^*+/+*^* - Clozapine 0 mg/kg:Htr*2a^*-/-*^*	2383.313	-2281.76	
				Clozapine 0 mg/kg:Htr2a*^*+/+*^* - Clozapine 5 mg/kg:Htr2a*^*+/+*^*	-414.784	-4881.25	
				Clozapine 0 mg/kg:Htr2a*^*+/+*^* - Clozapine 20 mg/kg:Htr2a*^*+/+*^*	-1362.27	-6027.34	
				Clozapine 0 mg/kg:Htr*2a^*-/-*^* - Clozapine 5 mg/kg:Htr*2a^*-/-*^*	1852.895	-2613.57	
				Clozapine 0 mg/kg:Htr*2a^*-/-*^* - Clozapine 20 mg/kg:Htr*2a^*-/-*^*	-265.062	-4930.13	
				Clozapine 5 mg/kg:Htr2a*^*+/+*^* - Clozapine 5 mg/kg:Htr*2a^*-/-*^*	4447.76	189.1545	
				Clozapine 20 mg/kg:Htr2a*^*+/+*^* - Clozapine 20 mg/kg:Htr*2a^*-/-*^*	3480.52	-1184.55	
Extended Data Fig. 2-1*A*	Not normal	Mann–Whitney test	Orb	Clozapine 5 mg/kg, male			6
				Clozapine 5 mg/kg, female			6
	Normal	*t* test with Welch’s correction	Pir	Clozapine 5 mg/kg, male	170.4	282.1	
				Clozapine 5 mg/kg, female	80.06	433.9	
	Not normal	Mann–Whitney test	Cpu	Clozapine 5 mg/kg, male			5
				Clozapine 5 mg/kg, female			3.25
	Not normal	Mann–Whitney test	Cg	Clozapine 5 mg/kg, male			4
				Clozapine 5 mg/kg, female			4.8
	Not normal	Mann–Whitney test	Vpm	Clozapine 5 mg/kg, male			6.25
				Clozapine 5 mg/kg, female			5
	Normal	*t* test with Welch’s correction	Ven	Clozapine 5 mg/kg, male	1810	3086	
				Clozapine 5 mg/kg, female	1728	2991	
Extended Data Fig. 2-1*B*	Normal	*t* test with Welch’s correction	Orb	Clozapine 0 mg/kg, male	43.25	82.37	
				Clozapine 0 mg/kg, female	33.38	60.78	
	Normal	*t* test with Welch’s correction	Pir	Clozapine 0 mg/kg, male	51.08	105.4	
				Clozapine 0 mg/kg, female	49.77	91.82	
	Not normal	Mann–Whitney test	Cpu	Clozapine 0 mg/kg, male			3.375
				Clozapine 0 mg/kg, female			6.3
	Normal	*t* test with Welch’s correction	Cg	Clozapine 0 mg/kg, male	42.15	103.2	
				Clozapine 0 mg/kg, female	39.36	94.81	
	Not normal	Mann–Whitney test	Vpm	Clozapine 0 mg/kg, male			7.4
				Clozapine 0 mg/kg, female			5.857
	Normal	*t* test with Welch’s correction	Ven	Clozapine 5 mg/kg, male	196.1	298.8	
				Clozapine 5 mg/kg, female	176.8	319.8	
Extended Data Fig. 2-1*C*	Normal	One-way ANOVA	Orb	Vehicle - Clozapine 5 mg/kg	47.26361	-237.813	
				Vehicle - Clozapine 20 mg/kg	-93.267	-328.661	
	Normal	One-way ANOVA	Pir	Vehicle - Clozapine 5 mg/kg	30.72838	-159.372	
				Vehicle - Clozapine 20 mg/kg	-89.141	-243.332	
	Normal	One-way ANOVA	Cg	Vehicle - Clozapine 5 mg/kg	37.98281	-263.291	
				Vehicle - Clozapine 20 mg/kg	-35.4183	-304.886	
	Normal	One-way ANOVA	Vpm	Vehicle - Clozapine 5 mg/kg	-31.2811	-302.009	
				Vehicle - Clozapine 20 mg/kg	-173.181	-410.795	

## Synthesis

Reviewing Editor: Karen Szumlinski, University of California at Santa Barbara Decisions are customarily a result of the Reviewing Editor and the peer reviewers coming together and discussing their recommendations until a consensus is reached. When revisions are invited, a fact-based synthesis statement explaining their decision and outlining what is needed to prepare a revision will be listed below. The following reviewer(s) agreed to reveal their identity: Bryan Roth, Andrea de Bartolomeis.

Upon consultation with two experts in the neuropharmacology of antipsychotic medications, I am pleased to inform you that we all agree that your report contains some very new and interesting information that are worthy of publication in eNeuro. The paper is very straight-forward and well-written. However, both reviewers had relatively minor comments that we all feel would improve the manuscript. As such, I have issued the decision of Revise-Re-review. Please see below for the specific comments of each reviewer.

Reviewer 1:

This is an interesting paper which provides new data on cellular targets of typical and atypical antipsychotic drugs. The authors find that genetic deletion of 5-HT2A receptors does not alter the c-fos expression in neurons but does in epindymal cells. This is interesting!

The authors should mention prior studies by others that genetic deletion of 5-HT2A does not alter the behavioral responses to clozapine (Yadav et al, 2011;

Bortolozzi A, Masana M, Díaz-Mataix L, Cortés R, Scorza MC, Gingrich JA, Toth M, Artigas F.

Int J Neuropsychopharmacol. 2010 Nov;13(10):1299-314) while others are dependent on 5-HT2a (Neuropsychopharmacology. 2012 Dec;37(13):2747-55. doi: 10.1038/npp.2012.139).

Reviewer 2:

The authors used the transgenic ‘FosTRAP’ mice , where a fluorescent reporter marks the cells responsive to the stimulus of interest, to detected the brain region, where c-fos expresion is induce or increased by antipsychotics.

Despite the fact that the results replicated what already known by ex vivo studies, the novelty of the methodology applied in vivo to the search of brain region impacted by antipsychotics and activating c-fos is worth to be disseminated.

My major criticism is that despite being a straightforward study with a quite good amount of work this report compared to other previous studies adds, in my opinion, the real new finding is the activation of cfos at level the ependyma in vivo.

However the opportunity to address this result in terms of putative translational significance is only in part considered by the authors.

First it will relevant to have few lines in the manuscript on the role of ependyma in schizophrenia pathophysiology in order to set the scenario of this intriguing brain region in the overall action of antipsychotics.

Second, It will be of interest to know which receptor(s) is targeted by antipsychotics in order to induce/increase c-fos expression in ependyma

Third it would interesting to introduce an hypothesis of the relevance, if any, of this brain activation, for the antipsychotic treatment.

Minor points

Introduction

The authors state

Even today antipsychotics are the preferred treatment for a number of mood disorders (Blier, 2005; Cookson, 2008; Leucht et al., 2009; McDougle et al., 2000).

Antipsychotics are used in the treatment of mood disorder however the first choice for mania and bipolar disorder are the mood stabilizers and for depression the antidepressants, being the antipsychotics mainly used for mania or (in the case of few antidepressants)in augmentation with antidepressant for treatment resistant or poorly responsive depression.
